# Simple and Efficient AlN-Based Piezoelectric Energy Harvesters

**DOI:** 10.3390/mi11020143

**Published:** 2020-01-28

**Authors:** Imrich Gablech, Jaroslav Klempa, Jan Pekárek, Petr Vyroubal, Jan Hrabina, Miroslava Holá, Jan Kunz, Jan Brodský, Pavel Neužil

**Affiliations:** 1 Central European Institute of Technology, Brno University of Technology, CZ-61600 Brno, Czech Republic; ja.klempa@gmail.com (J.K.); pekarek@vutbr.cz (J.P.);; 2 Department of Microelectronics, Faculty of Electrical Engineering and Communication, Brno University of Technology, CZ-61600 Brno, Czech Republic; 3 Department of Electrical and Electronic Technology, Faculty of Electrical Engineering and Communication, Brno University of Technology, CZ-61600 Brno, Czech Republic; vyroubal@feec.vutbr.cz; 4 Institute of Scientific Instruments, Czech Academy of Sciences, CZ-61264 Brno, Czech Republic; hrabina@isibrno.cz (J.H.); hola@isibrno.cz (M.H.); 5 Department of Control and Instrumentations, Faculty of Electrical Engineering and Communication, Brno University of Technology, CZ-61600 Brno, Czech Republic; kunzj@feec.vutbr.cz; 6 Department of Microsystem Engineering, School of Mechanical Engineering, Northwestern Polytechnical University, Xi’an 710072, China; pavel.neuzil@gmail.com

**Keywords:** AlN, micro-electro-mechanical systems (MEMS) cantilever, complementary metal oxide semiconductor (CMOS) compatible, energy harvesting, high performance

## Abstract

In this work, we demonstrate the simple fabrication process of AlN-based piezoelectric energy harvesters (PEH), which are made of cantilevers consisting of a multilayer ion beam-assisted deposition. The preferentially (001) orientated AlN thin films possess exceptionally high piezoelectric coefficients *d*_33_ of (7.33 ± 0.08) pC∙N^−1^. The fabrication of PEH was completed using just three lithography steps, conventional silicon substrate with full control of the cantilever thickness, in addition to the thickness of the proof mass. As the AlN deposition was conducted at a temperature of ≈330 °C, the process can be implemented into standard complementary metal oxide semiconductor (CMOS) technology, as well as the CMOS wafer post-processing. The PEH cantilever deflection and efficiency were characterized using both laser interferometry, and a vibration shaker, respectively. This technology could become a core feature for future CMOS-based energy harvesters.

## 1. Introduction

Energy harvesting has recently attracted significant attention as a key power source where changing batteries in applications is not practical, or in low-power autonomous sensors and micro-devices, as a replacement of electrochemical batteries.

Several methods of harvesting ambient energies have been investigated, including solar energy, wind, flowing water, waste heat, electromagnetic waves, or vibrations [1,2]. However, most of them require the outside environment. The utilization of mechanical vibrations represent a suitable alternative for any environment, including indoors, as well as low-power autonomous sensors and microdevices [3].

Electrostatic and electromagnetic induction, and piezoelectricity can all typically be exploited as transducing mechanisms to convert mechanical energy into electrical [4]. However, from these, piezoelectric energy harvesters (PEHs) exhibit high-energy density and are, therefore, more suitable for practical applications [5]. Moreover, piezoelectric materials have an inherent capability to directly convert mechanical stress/strain energy into electrical energy, therefore, such devices are compact and possess simpler designs, compared to their electromagnetic and electrostatic counterparts. Furthermore, such devices can be fabricated by micromachining techniques and directly integrated into monolithic, micro-electro-mechanical systems (MEMS) [6].

Numerous piezoelectric materials were investigated for energy harvesting in MEMS applications, but the most commonly used are ZnO [7], lead zirconate titanate (PZT) [8,9], polyvinylidene fluoride (PVDF) [10], and AlN [11]. In particular, AlN, prepared by sputtering, can be implemented in standard complementary metal oxide semiconductor (CMOS) technology, as well as the CMOS wafer post-processing [12], thereby, enabling the integration of PEH with active devices. Other piezoelectric materials such as PZT, ZnO, and PVDF possess contamination risks for CMOS processing lines [13], while AlN, deposited by the metal-organic chemical vapor deposition (MOCVD) technique, requires high temperature, which prohibits its integration with CMOS devices.

Sputtered AlN is a promising material for PEH applications, due to low-temperature preparation, unique physical properties (such as a high thermal stability, with a melting point of ≈2100 °C and piezoelectric effect up to temperatures of ≈1150 °C; high longitudinal velocity of ≈11,000 m·s^−1^; and wide band gap of ≈6.2 eV), high level of mechanical stiffness, and good piezoelectric and dielectric properties [11]. 

The single side clamped cantilever structure, due to its simple design and fabrication, is a convenient device to characterize properties of PEHs: It can produce large mechanical strain within the piezoelectric layer with its vibrations [14]. The amplitude of generated piezoelectric voltage and power depends on the device’s working frequency, as well as the value of induced strain. The first resonance frequency (*f*_r_) of a cantilever is the lowest vibrational mode, exhibiting the highest achievable strain and displacement. The goal of the harvester design is to operate at the *f*_r_ to achieve maximum power output.

Normalized power density (*NPD*), together with output power (*P*) and frequency range, also known as bandwidth (*BW*), are the most widely used metrics to evaluate the performance of PEH [15]. They enable the comparison of different PEHs and provide necessary information for figure of merit (*FoM*) calculations [16],

(1) FoM=NPD×BW

where the *NPD* is defined as *P* divided by the effective volume (*V*) and the square of the input acceleration (*A*),

(2) $$NPD=\frac{P}{V\cdot A^{2}}$$

and where *BW* is defined as,

(3) $$BW=f_{2}-f_{1}$$

where *f*_1,2_ are half-power, cut-off frequencies, also known as full width at half maximum (FWHM). Bandwidth comparison is often complicated, as its definition is not standardized: Sometimes it is defined by frequencies at FWHM of the spectrum, by 1 dB or 3 dB bandwidth, or the data is not available.

Fabrication of PEHs is notoriously complex, requiring five or more lithography steps, in addition to expensive silicon-on-insulator (SOI) substrates. Here we show a simple method to prepare PEH with a high value of piezoelectric coefficients of (7.33 ± 0.08) pC∙N^−1^, using low temperature ion-assisted deposition, making it fully CMOS-compatible, including the CMOS wafer post-processing.

## 2. Materials and Methods

### 2.1. Chip Design and Fabrication

Technology flow and layout were designed to allow all thin films to be deposited sequential inside the sputtering system. This is a key feature, as it enables the deposition of all layers without breaking the vacuum, thereby, resulting in high-quality layers and good adhesion between them, while eliminating contamination and achieving a high performance in the piezoelectric layer.

**Figure 1:**
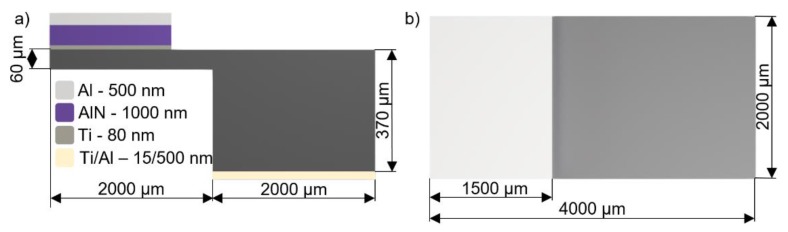
Layer composition and their dimensions of piezoelectric energy harvesters (PEH) (not to scale): (**a**) side view; (**b**) top view.

The beam and proof mass had dimensions of 2000 µm × 4000 µm and 2000 µm × 2000 µm, respectively. A piezoelectric layer between two electrodes, with dimensions of 1500 µm × 2000 µm, is placed on one end close to the fixed edge (Figure 1).

We had to remove a relatively large area of Si substrate, as its direct etching would result in a high loading factor and etching process instability. We designed 40 µm wide trenches around the PEH beams, on the both sides of the substrate. Once the deep reactive ion etching (DRIE) from both sides was completed, the area surrounded by the trenches fell away from the substrate without the necessity to etch it, as demonstrated in the design in supplementary materials of previously published work [17]. A large-area substrate removal around the PEH beams led to their unobstructed movement.

**Figure 2:**
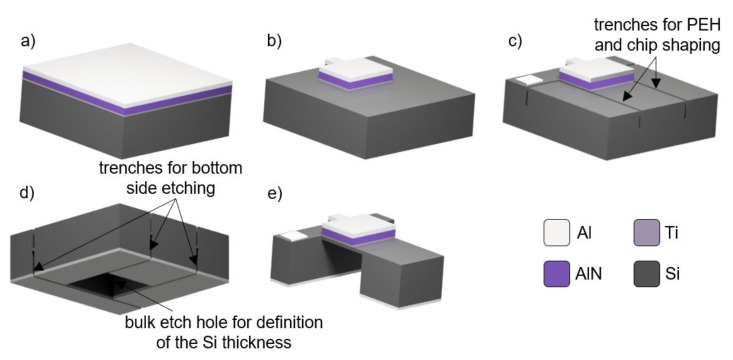
Fabrication process flow (not to scale): (**a**) Deposited layers on top side of Si substrate; (**b**) patterning of Ti/AlN/Ti/Al structure; (**c**) top-side trench etching using deep reactive ion etching (DRIE) method; (**d**) metallization followed by back-side etching causing separation of chips; (**e**) back-side etching, using DRIE method to form final structure.

The fabrication was conducted using double-side polished Si (100) N-doped wafers with a diameter of ≈100 mm, thickness of ≈370 µm, and resistivity of <0.005 Ω∙cm. We deposited all sequential thin films, layer by layer (Figure 2a), using an ion-beam assisted deposition (IBAD) instrument, without breaking the vacuum between depositions. The wafers were loaded into the IBAD instrument and the system was evacuated to a base pressure of ≈9 × 10^−7^ Pa. Wafers were then pre-cleaned using a secondary ion-beam source with Ar plasma with 30 V beam voltage (*BV*), for a duration of 300 s. We then deposited ≈80 nm of Ti, serving as a seed layer for consequent AlN (001) deposition, as well as an electrical connection between the AlN and Si substrate. We activated the primary Kaufman ion-beam source, using a *BV* of 200 V, resulting in a (001) oriented layer of Ti [18,19]. This was followed with a change in the *BV* to 400 V and the addition of N_2_ to the primary ion-beam source, with a ratio of 1:1 to Ar. In addition, we employed the secondary ion-beam source for substrate bombardment, using N_2_ plasma at a *BV* = 30 V and performed reactive sputtering of highly (001) oriented AlN from the Al target, to achieve the desired thickness of ≈1000 nm [20].

Finally, we halted the secondary ion-beam source and N_2_ from the primary source and deposited the Al layer using a *BV* of 900 V, achieving an Al thickness of ≈500 nm, suitable for subsequent wire-bonding.

The wafers were then subjected to just three lithography steps. The first lithography step was completed using positive photoresist (PR), with a desired thickness of ≈1.4 µm, to define the shape of the top electrode, piezoelectric layer, and underneath Ti in a single stage. It was followed by reactive ion etching (RIE) with combined Cl_2_ and BCl_3_ gases, using an optical spectrometer to monitor the etching process (Figure 2b). After etching and PR removal, we performed the second lithography, using PR with a thickness of ≈10 µm to define the PEH shape and the DRIE process to etch ≈40 µm wide and ≈150 µm deep trenches around them (Figure 2c). Following this, we removed the thick PR and spin-coated, front side of the Si substrate with a standard PR to protect it; this subsequently deposited Ti and Al with a thickness of ≈15 nm, and ≈500 nm, respectively, on the back side of the Si wafer, forming backside electrode contact. We conducted backside lithography, with front-to-back alignment, using thick PR and etched both metals using Cl_2_/BCl_3_-based reaction ion etching (RIE). We continued with DRIE, through the Si substrate, until the inner parts and chips were separated from each other (Figure 2d). The thickness of PEH in areas without the proof mass was ≈50 µm. The chips were mounted individually on a supporting base of Si substrate, using a drop of Fomblin^®^ oil, and etched using the DRIE method, until we reached the desired thickness (Figure 2e) of a few tens of µm. The proof mass thickness was ≈370 µm, allowing us to fabricate the PEH with a high mass-to-volume ratio. The residual PR and Fomblin^®^ were then removed with O_2_ plasma.

**Figure 3:**
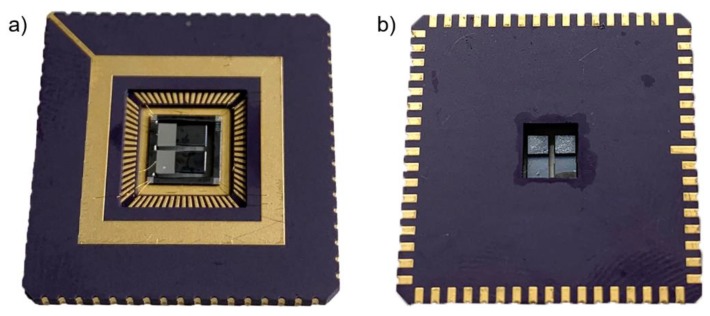
Fabricated PEH mounted in LCC68: (**a**) top view; (**b**) bottom view showing hole and PEH mass.

Finally, we cut a 4.5 × 4.5 mm^2^ hole, using an yttrium aluminum garnet (also known as YAG) laser into the center of the leadless chip carrier with 68 pads (LCC68). The individual chips were then mounted using silver conductive paste into the LCC68 (Figure 3a). The mounted chips had free vertical movement within the mass of the package (Figure 3b).

### 2.2. X-Ray Characterization

**Figure 4:**
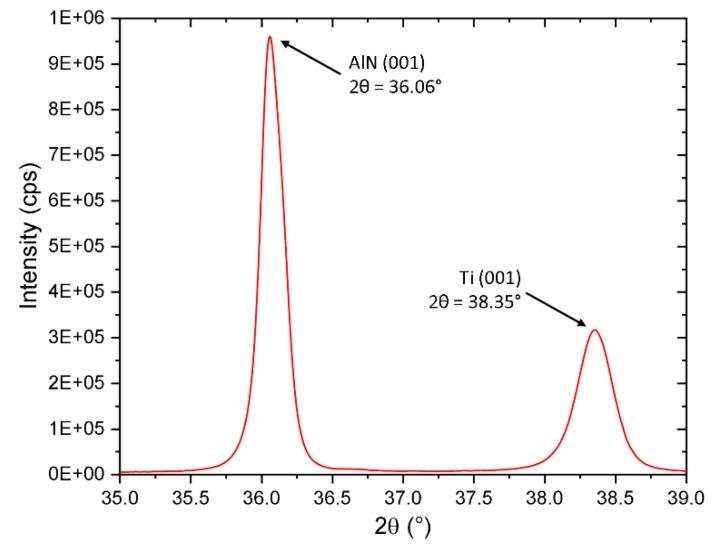
X-ray diffractogram determined using Brag-Brentano setup showing 2*θ* peak positions of Ti (001) ≈38.35° and AlN (001) ≈36.06°.

Deposited Ti and AlN layers were residual stress-free, which was determined from wafer curvature measurement. We also conducted the X-ray measurement using Bragg-Brentano setup to determine corresponding peak positions (Figure 4) for 2*θ* ≈38.35° for Ti (001) and 2*θ* ≈36.06° for AlN (001). These peaks positions also perfectly fit residual stress-free values determined from lattice parameters we published earlier [18,20]. Such prepared (001) oriented AlN exhibits a high value of piezoelectric coefficient *d*_33_ of (7.33 ± 0.08) pC∙N^−1^ along c-axis.

### 2.3. Finite Element Simulation

**Figure 5:**
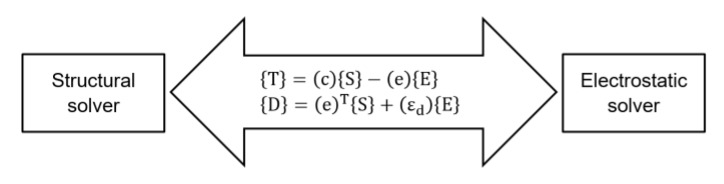
Scheme of coupled solution for electrostatic and structural solver, employing piezoelectric matrix.

We performed finite element method (FEM) analyses of single clamped PEH using the ANSYS^®^ Workbench with the Piezo and MEMS module. The model geometry was formed with a SOLID186 and SOLID226 3D element with a 20-node coupled-field, solid supporting piezoelectric analysis [21]. We performed coupled solution using an electrostatic and structural solver (Figure 5) via the piezoelectric matrix where {T} is the stress matrix, (c) is the elastic stiffness matrix, {S} is the elastic strain vector, (e) is the piezoelectric matrix, {E} is the electric field intensity vector, {D} is the electric flux density vector, and (ε_d_) is the dielectric permittivity matrix.

Once we built the model, we performed modal analysis to determine resonance frequencies of the entire system. It was followed by a harmonic analysis used to determine PEH behavior under an external force, using the results from the modal analysis as boundary conditions. Then we applied the excitation voltage on electrodes to determine the displacement of PEH and compare it with experimental results.

**Figure 6:**
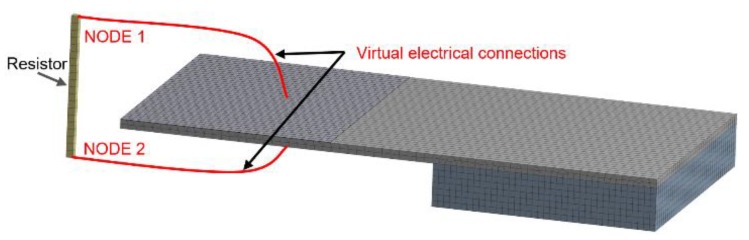
Scheme of PEH model with electrically connected *R*_L_.

Following this, we added a load resistor (*R*_L_) into the model, applying CIRCU94 circuit 2-node beam elements using ANSYS^®^ parametric design language (also known as APDL) commands, and examined the dependence of generated power (*P*_S_) on the amplitude of acceleration and the value of parallel connected *R*_L_ (Figure 6).

This task was realized as a combined analysis, involving the mechanics of a rigid body with a link to a piezoelectric effect (or the inverse piezoelectric effect) and the provision of a bond to an electrical circuit simulating *R*_L_.

## 3. Results and Discussion

We characterized PEHs using two methods to validate their parameters and compare them to the FEM simulations from the ANSYS^®^ Workbench.

### 3.1. Laser Interferometer Characterization

**Figure 7:**
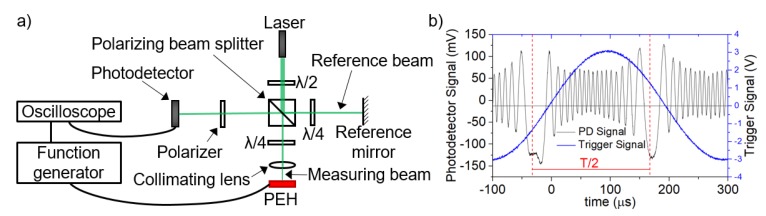
Interferometric measurement: (**a**) setup; (**b**) oscilloscope electrical signal.

We chose the laser interferometer measurement as the first method for resonance frequency and displacement determination (Figure 7a). We used a diode-pumped solid-state laser with single longitudinal-mode operation and output wavelength (λ) of ≈532 nm. The interferometric setup employs a classic Michelson arrangement. Illuminating light enters the polarizing beam-splitter, where it is split into two beams: The measuring beam passes to the sample, where it is reflected with phase shift into the beam splitter and on to the detector; the second (reference) beam is reflected from a fixed-reference mirror. Both beams interfere at the detector, which converts the optical signal of the incident beams into an electrical signal that is displayed on an oscilloscope (Figure 7b). The voltage power supply, with an alternate current (*V*_AC_) and sinusoidal signal, was applied on PEH electrodes. Displacement of the PEH on z-axis (*D*_Z_) is proportional to the number of interferometric fringes between minimum and maximum amplitudes of the exciting signal, multiplied by λ/2.

**Figure 8:**
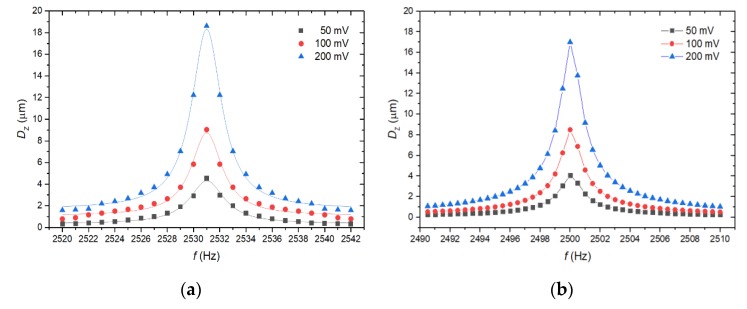
(**a**) Measured *D*_Z_ at *f*_r_ = 2520 Hz with various *V*_AC_. (**b**) The results of FEM simulations for the same *V*_AC_ bias.

The *V*_AC_ with a sinusoidal signal was applied on PEH electrodes. We adjusted *V*_AC_ within a range of 0.05 V to 0.2 V. We observed the first *f*_r_ at ≈2520 Hz, which agrees with results obtained by simulations with the corresponding value of ≈2500 Hz. The *D*_Z_ obtained from the measurement at *f*_r_ varied within a range of ≈4.5 µm to ≈18.2 µm for different *V*_AC_, this corresponded with measurement data for a range of ≈4.1 µm to ≈17.1 µm for the same *V*_AC_ values. The dependence of measured *D*_Z_ on frequency is shown in Figure 8a. The measured values of *D*_Z_ correlate with values determined in FEM analyses in Figure 8b.

### 3.2. Vibrational Characterization

**Figure 9:**
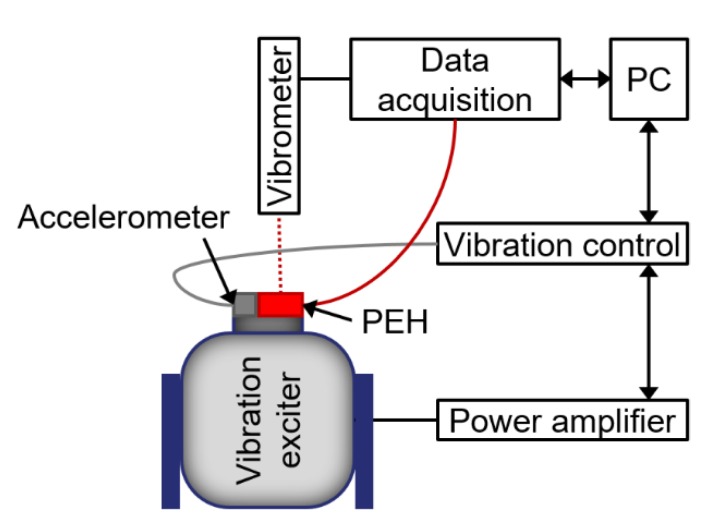
Measurement setup, based on vibration excitation of PEH for determination of *P*_M_.

Next, we characterized generated power (*P*_M_) using an automatized measurement system (Figure 9) [22].

We placed the PEH on a table with controlled sinusoidal vibrations of specific amplitude and frequency, near to the first *f*_r_, as extracted from the previous interferometric measurement. The test system was able to determine the *f*_r_ value, thus, we performed the measurement in proximity to this value. The measurement started by connecting an *R*_L_ to the PEH, while the stage was vibrating. Once the amplitude of the vibrations was stabilized, we recorded voltage across *R*_L_ (*V*_RL_) amplitude, together with the free end of PEH displacement. The power output of the harvester was calculated from the known *R*_L_ and *V*_RL_ [23]. This procedure was then performed repeatedly for all pre-set combinations of *R*_L_, frequencies, and amplitudes.

**Figure 10:**
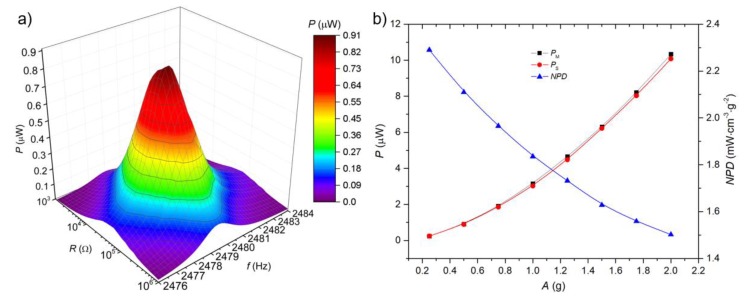
(**a**) Power spectra of measured PEH near *f*_r_ = 2480 Hz with constant *A* ≈ 0.5 g, (**b**) dependence of maximum generated *P*_S_ and *P*_M_ and calculated value of *NPD* on *A*.

We observed a slight shift in *f*_r_ in comparison to the *f*_r_ determined during interferometric measurement. We changed the *R*_L_ in the range from 100 Ω to 1 MΩ, with a logarithmic stepping for frequencies in a range of 2476 Hz to 2484 Hz with constant *A* ≈ 0.5 g. The optimized *R*_L_ value of ≈67.56 kΩ was found for maximal generated *P*_M_ of ≈0.91 µW at *f*_r_ with *A* ≈ 0.5 g (Figure 10a).

We also determined the dependence of *P*_M_ on *A* for maximized *R*_L_. The obtained values showed a remarkable correlation between predicted parameters from FEM analyses and the one of the fabricated device (deviation lower than 1%). We observed values of *P*_M_ in the range of ≈0.25 μW to ≈10.33 μW for the *A* in the range of ≈0.25 g to ≈2 g at *f*_r_ (Figure 10b).

Additionally, we identified a difference of ≈1.6 % between the *f*_r_ determined from interferometric and vibrometer measurements.

We subsequently calculated *NPD* and *BW* values from the results obtained in the last experiment. The *NPD* was determined (according to the Equation (2) with an assumption that the effective volume was ≈1.72 × 10^−3^ cm^3^) having a value in a range of ≈2.3 mW∙ cm^−3^∙g^−2^ to ≈1.5 mW∙cm^−3^∙g^−2^ for *A* in range of 0.25 g to 2 g (Figure 10b).

Such values of *NPD* are ≈2–10 times higher in comparison to previously published *NPD* [24,25,26,27,28]. The *BW* value was also determined for frequencies at FWHM of the spectrum, at a constant of *A* ≈ 0.5 g and reached a value of ≈2.8 Hz.

## 4. Conclusions

This work presents a simplified method of PEH fabrication at a low temperature, using just three lithographical steps, and without the necessity of using costly SOI wafers, which dramatically reduces the manufacturing timeframes and costs. This method also allows the control of the thickness of the PEH layer from tens to hundreds of micrometers. We characterized the PEH properties using interferometric measurements, and automated power measurement using a vibration exciter. First, we applied *V*_AC_ on PEH electrodes to determine *f*_r_ and *D*_Z_, comparing them with FEM analyses to verify the model. Next, we characterized *P*_M_ at *f*_r_ and determined the optimized *R*_L_ value to maximize *P*_M_ at ≈67.56 kΩ. The PEH generated *P*_M_ in a range of ≈0.25 µW to ≈10.33 µW for *A* in a range of ≈0.25 g to ≈2 g, respectively. The determined *NPD* and *BW* values performed better than those of previously published studies, making the proposed technology highly promising for the future development of CMOS-compatible piezoelectric harvesters.
